# Case of Intermittent Inflammation of the Brain, Relieved by Copious Blood-Letting during the Paroxysms, and Large Doses of Opium at Bed-Time

**Published:** 1818-12

**Authors:** E. C. Parkinson


*For the London Medical and Physical Journal.*

### *Case of intermittent Inflammation of the Brain, relieved by copious Blood-letting during the Paroxysms, and large Doses of Opium at Bed-time;* by E. C. Parkinson, Esq.

ON the 14th of May, 1818, I was requested to visit Elizabeth Martin, a stout woman, about 45 years of age, who complained of intolerable pain in the head, principally at its back part, lassitude, weariness of her limbs, impatience of strong light and sounds, intense thirst, and delirium. She had, for several days previously, complained of considerable pain in the head, but considered it what she had been accustomed to—the head-ache. On the morning of the 14th of May, she was free from pain, and, as she conceived, was perfectly well; but, about four o'clock in the afternoon, the pain returned with increased violence, followed by a rigor, which continued nearly an hour, succeeded by great heat, hard wirey pulse—108 in the minute, delirium, pupils contracted, the face flushed, eyes slightly inflamed.

I considered this a sufficiently-marked case of inflammation in the encephalon to justify me in the adoption of the most prompt and powerful means of reducing it, notwithstanding it might be objected to by some, who were aware that what is usually called typhous fever then prevailed in the neighbourhood; which, give me leave to add, was, as it is always, only symptomatic of inflammation.

I immediately bled her *ad syncopen*; directed a bolus, containing six grains of hydrargyri submurias, to be taken immediately; drinking three table-spoonfuls of the following mixture every three hours, till the bowels were well emptied:—℞. Magnes. sulphat. ℥ij; Rhæi pulv. ʒi; Aquæ fontan. ℥vij. M. f. mistura. After the operation, I directed the following opiate:—℞. Tinct. opii, gtt. lx.; Mist. camphor, ℥ij. M. f. haustus horâ somni sumendus. I entirely prohibited solid food and strong drinks, allowing her to take plentifully of toast-and-water and thin gruel.

The pulse continued more open, and softer, after recovery from syncope; the opening medicines operated briskly, and the opiate procured a good night's sleep.

On the 15th, in the morning early, she was free from pain in the head; perspired freely; very much reduced by yesterday's treatment; pulse 65, very soft and open; urine highly coloured, depositing a lateritious sediment.

Allowed cooling sippings only, and a rigid abstinence from wine or fermented liquors.—℞. Antimon. tartart. gr. ij.; Sacch. alb. ℈j.; Potassæ supertart. ℈j. M. et distribue in partes vi. quarum unam horâ quartâ quaque sumat in cochl. larg. iij. Misturæ insequentis.—℞. Magnes. sulphat. ʒvj.; Infus. rosæ, ℥viij. M. f. mistura. The opening mixture to be had recourse to early the next morning; the opiate to be given this evening at bed-time.

16th.—Paroxysm returned, but much slighter, and not before eight o'clock in the evening. The opening mixture had operated well in the fore-part of the day. The antimonials were continued, and the opiate repeated at bed-time. Sweated freely, and slept well after the paroxysm.

17th.—Broth allowed. Persistat in usu pulverum et misturæ. The opiate to be repeated at bed-time if the paroxysm should return, but not otherwise.

18th.—No paroxysm yesterday; therefore the opiate not taken. Tongue clean, pulse regular and natural; more nourishing diet allowed.

She continued well till the 26th, when she experienced a return of pain in the head. Bleeding, the opening medicines, and the opiate, were repeated.

27th.—In the morning, free from complaints; at 3 P. M. a paroxysm. The opiate repeated at bed-time; opening medicines in the morning.

28th.—Paroxysm at 4 P. M.—℞. Antimon. tartar. gr. ij; Sacch. alb. ʒj. tere bene simul, inter terendum addendo—Pulv. cinchon. ℥j. M. et divide in pulveris xij. sumat i. horâ 3tia. quaq. paroxysmo absente, in poculo lactis, cremorâ priùs ademptô.

29 and 30.—Very slight paroxysms; after which she had no return.

Perhaps there may not appear novelty enough in the above case to render it worthy of the attention of your readers; yet it bears a strong resemblance to all those diseases which prevailed in the neighbourhood, denominated typhous fever, in many instances proving fatal when treated with stimulants, but in no one instance either ending fatally or of long duration when treated after the manner of the case above related,—that is, free bleeding in the paroxysms; cathartics, antimonials, and ample doses of opium at bed-time; watching for a favourable opportunity of putting off the succeeding paroxysm by the bark.

Let me observe, all fevers are symptomatic of some local inflammation. This doctrine I have been taught by my father, and experience has confirmed it. Not that the local inflammation is always in the head; it is very commonly in the abdomen, in the thorax, and not unfrequently on the external surface, described by the patient as weariness of the limbs, rheumatism, &c. Situated, however, where it may, it must be either of the remittent type or of the intermittent type, until it obtains such a degree of intensity as to give it the progressive type,—that is, proceeding to gangrene of the parts principally aggrieved, and the exhaustion of the whole of the vital energies, by a continued excess of exercise of the vital and animal functions above the limits of disease.

It must be remembered, that this relates only to the first order of inflammation—Hyperbiosis Diffusa, or inflammation of the first order of solids.

*Leicester; Oct.* 30, 1818.

